# Baló Concentric Sclerosis

**DOI:** 10.5334/jbsr.2914

**Published:** 2022-10-31

**Authors:** Kelly Di Dier, Marc Lemmerling

**Affiliations:** 1 AZ Sint-Lucas, BE; 2 AZ Sint Lucas, Ghent, BE

**Keywords:** concentric sclerosis, multiple sclerosis, demyelinating disease, MRI, neuroradiology

## Abstract

**Teaching Point:** Baló concentric sclerosis is a rare subtype of multiple sclerosis, characterized by a concentric layered mass on magnetic resonance imaging.

## Case History

**Figure 1:**
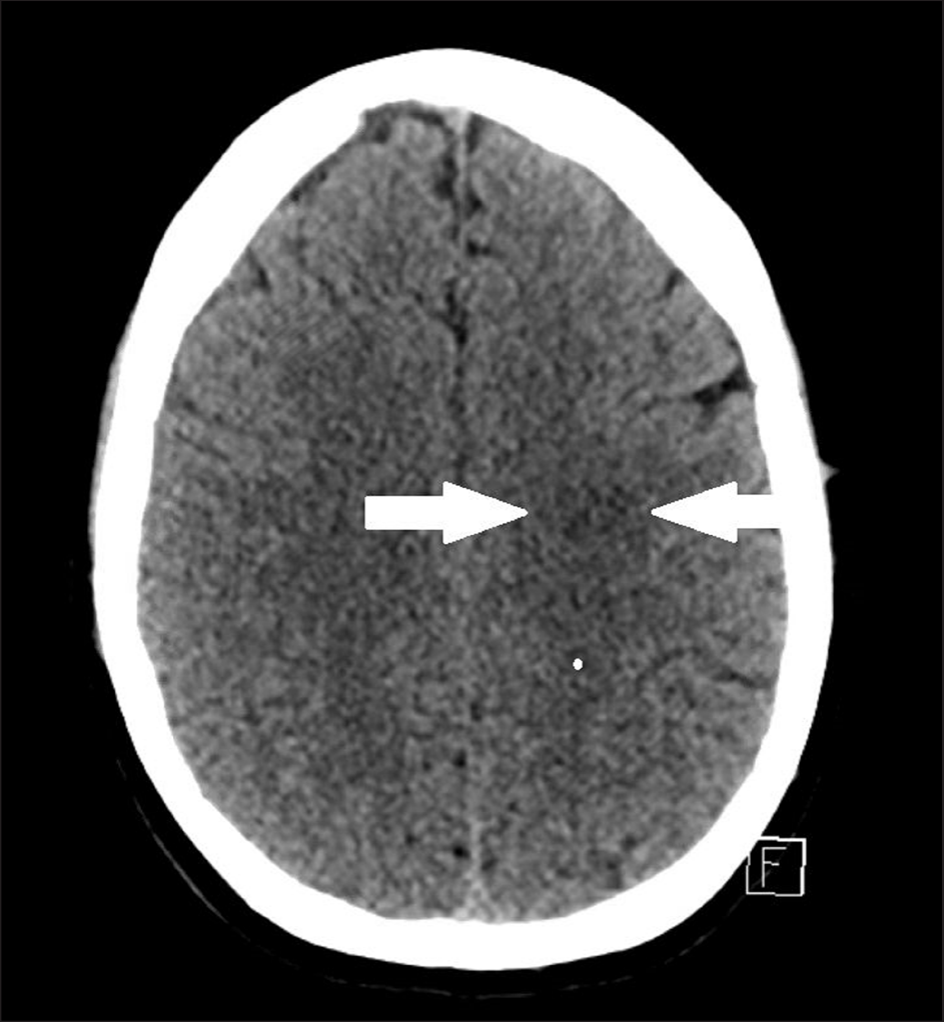


**Figure 2:**
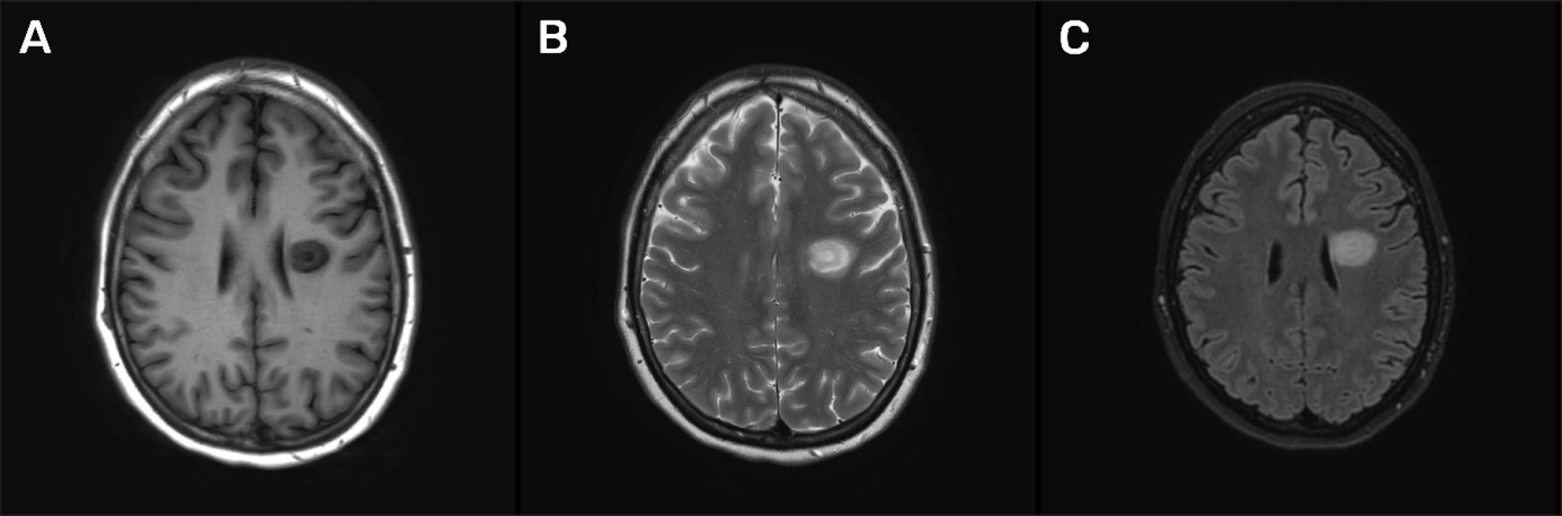


**Figure 3:**
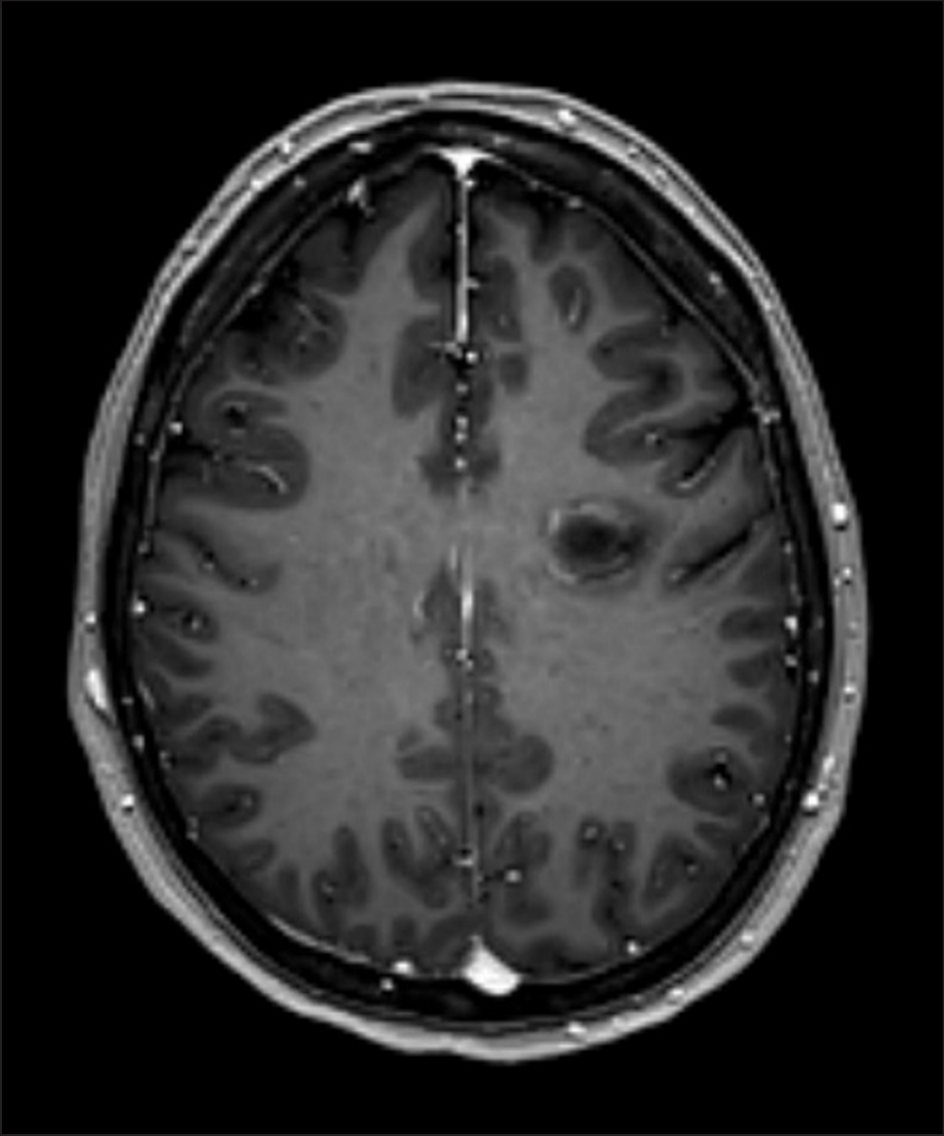


A 47-year-old woman presented at the emergency department with dysarthria and delayed speech. A plain computed tomography (CT) of the brain showed a hypodense lesion in the left frontal lobe, which was interpreted as an old infarction (Figure 1). On a subsequent magnetic resonance imaging (MRI) examination of the brain, a nodular mass was demonstrated in the left frontal lobe, characterized by concentric alternating signal intensity layers on the T1- and T2-weighted images (Figure 2A–2B, respectively), and less prominently on fat-suppressed FLAIR-weighted images (Figure 2C). No locoregional mass effect nor surrounding edema were seen. The outer border of the lesion moderately enhanced after intravenous injection of gadolinium (Figure 3). No other contrast enhancing lesions were identified. The diagnosis of active Baló concentric sclerosis was concluded.

## Comment

Baló concentric sclerosis is considered a variant/subtype of multiple sclerosis (MS). It is a severe monophasic demyelinating disease, mostly with fulminant course. This specific subtype of demyelination is rather rare in general, and occurs more among Asian population, whereas multiple sclerosis to the contrary is more prevalent in Caucasians [1].

The symptoms align with the broad spectrum of clinical manifestations seen in MS. Motor and/or sensible disorders can occur in an acute or subacute setting. Common complaints are headache, aphasia, cognitive impairment, changes in behavior, seizures, and the like [1].

Baló concentric sclerosis has a typical appearance on MRI. Concentric low to iso-intense layers are observed on T1-weighted images. That layering pattern is iso- to hyperintense on T2-weighted images. These imaging findings are well demonstrated in Figure 2. Active demyelination is marked by contrast enhancement, typically located in the peripheral ring of the lesion as shown in this case in Figure 3. In an early stage these findings only occur on MRI, making it the imaging modality of choice. In a later disease stadium, the typical layering pattern may vanish. Diagnosis should then be made with biopsy [1].

The underlying pathogenesis is not completely understood. It is suggested that the demyelination starts at a central core from which it expands centrifugally. Layers of demyelination are alternated with layers of preserved myelin/remyelination, causing the typical concentric pattern [1].

Differential diagnosis should be made with MS and its variants (Marburg variant, tumefactive MS), tumors, abscesses, acute disseminated encephalomyelitis [1], lymphoma, toxoplasmosis, and so on. Just as with MS, Baló concentric sclerosis is treated with corticosteroids. Despite its fulminant course, this pathology is not necessarily fatal.
